# Supplementary material for: Comparative multiomics analysis of cell physiological state after culture in a basket bioreactor
Source: Sci Rep. 2022 Nov 23;12:20161. doi: 10.1038/s41598-022-24687-4 (PMC9686226; doi:10.1038/s41598-022-24687-4)
Supplement: Supplementary file 1 — Supplementary Information 1. [file 41598_2022_24687_MOESM1_ESM.zip › raw data/Transcriptomic raw data/6.Enrichment/1.GO/TE1vsTC1/ALL/TE1vsTC1.all_GOdot.pdf]

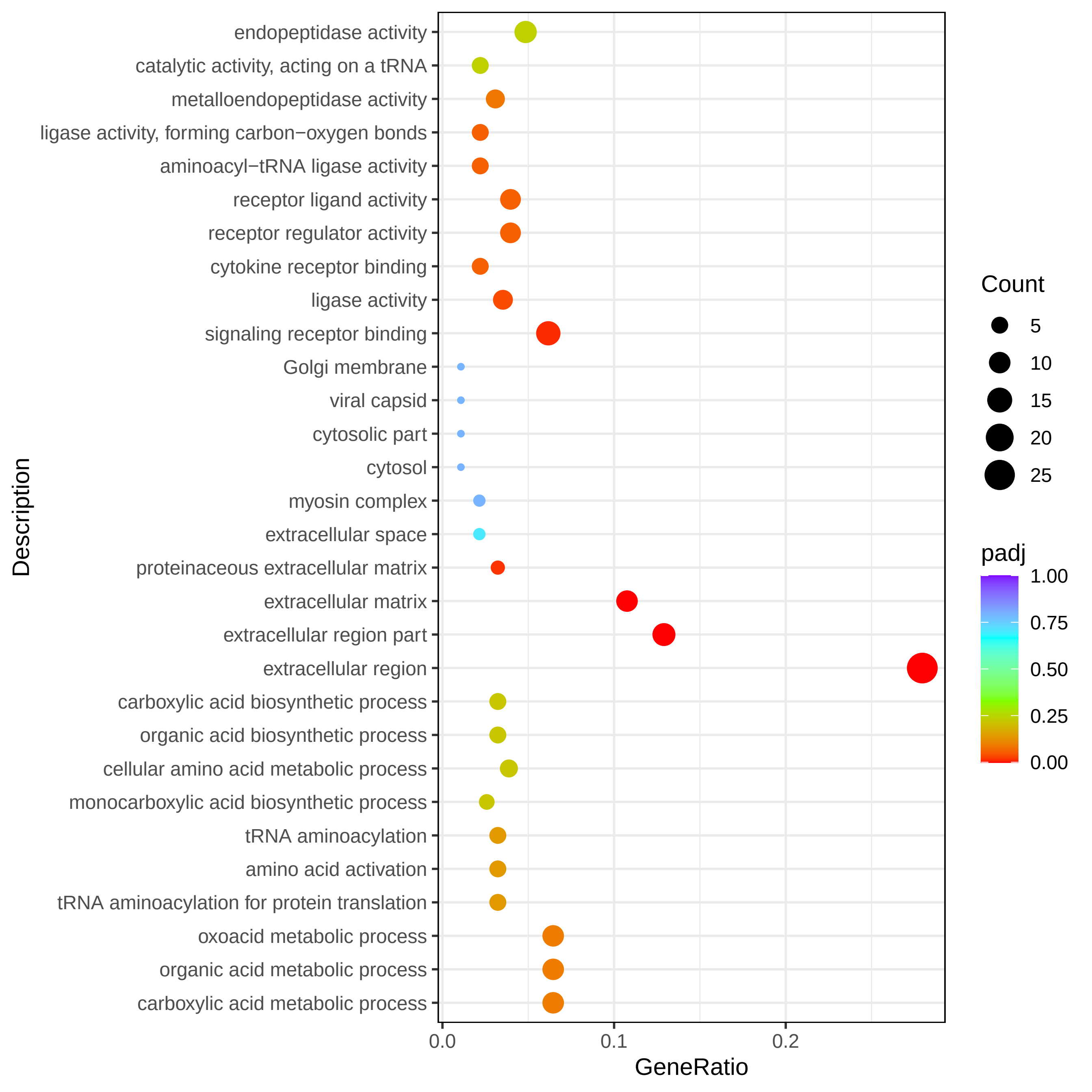
endopeptidase activity
catalytic activity, acting on a tRNA
metalloendopeptidase activity
ligase activity, forming carbon−oxygen bonds
aminoacyl−tRNA ligase activity
receptor ligand activity
receptor regulator activity
cytokine receptor binding
ligase activity
signaling receptor binding
Golgi membrane
viral capsid
cytosolic part
cytosol
myosin complex
extracellular space
proteinaceous extracellular matrix
extracellular matrix
extracellular region part
extracellular region
carboxylic acid biosynthetic process
organic acid biosynthetic process
cellular amino acid metabolic process
monocarboxylic acid biosynthetic process
tRNA aminoacylation
amino acid activation
tRNA aminoacylation for protein translation
oxoacid metabolic process
organic acid metabolic process
carboxylic acid metabolic process
Description
0.0
0.1
0.2
GeneRatio
Count
5
10
15
20
25
padj
1.00
0.75
0.50
0.25
0.00
